# Nonface-to-Face Visitation to Restrict Patient Visits for Infection Control: Integrative Review

**DOI:** 10.2196/43572

**Published:** 2023-11-28

**Authors:** Hyunwoo Jeong, Yonsu Choi, Heejung Kim

**Affiliations:** 1 College of Nursing Yonsei University Seoul Republic of Korea; 2 Department of Internal Medicine Nursing Seoul National University Hospital Seoul Republic of Korea; 3 Department of Surgical Nursing Seoul National University Hospital Seoul Republic of Korea; 4 Mo-Im Kim Nursing Research Institute Yonsei University Seoul Republic of Korea

**Keywords:** nonface-to-face visitation, visit restriction, infection control, patient, family

## Abstract

**Background:**

In the COVID-19 pandemic, a visit restriction policy for patients has been implemented in medical institutions worldwide and visits are being made using alternative communication technologies. This shift has also required the use of platforms to prevent negative consequences of these restrictions.

**Objective:**

The purpose of this review was to comprehensively explore nonface-to-face visits as an alternative during infection prevention and to synthesize the scientific evidence of their benefits and disadvantages.

**Methods:**

A comprehensive search was conducted via the PubMed, Embase, CINAHL, Cochrane, and Web of Science electronic databases; unpublished trials in the clinical trials register ClinicalTrials.gov; and Virginia Henderson International Nursing Library up to September 10, 2021. The search query was developed according to the guidelines of the Peer Review of Electronic Search Strategies and included keywords on the topics of telemedicine and visitation restrictions. The inclusion criteria were a nonface-to-face modality using telemedicine with family in a hospital setting, experimental and observational studies, and articles written in English. The exclusion criteria were inaccessible in full text, not related to patient or family involvement, mainly focused on the study protocol, or only discussing the pros and cons of telemedicine.

**Results:**

Overall, patients’ families experienced emotional distress due to restrictions on face-to-face visits. Nonface-to-face virtual visits compensating for these restrictions had a positive effect on reducing the risk of infection to the patient and the family. This further encouraged psychological and physical recovery and decreased psychological distress. However, nonface-to-face virtual technology could not replace the existence of actual families, and technical problems with networks and devices are reported as limitations.

**Conclusions:**

Ensuring the availability of technology and educating on the same in alignment with the characteristics of patients and their families, nonface-to-face virtual visits need to show more potential as an effective patient-centered treatment strategy based on more research and advanced practice.

## Introduction

Visiting family members in the hospital provides a chance for interaction and emotional stability to patients. Previous studies reported that open and flexible family visitation prevents patients’ delirium; reduces hospital days in the intensive care unit (ICU) [1,2]; and decreases anxiety, depression, loneliness, and distress levels [3,4]. In the case of newborns, parents have limited visits to the neonatal intensive care unit (NICU), which may increase health inequalities related to poor parental bond and postpartum depression [5,6]. Moreover, it is difficult for family members to receive family-centered care when visitation is restricted [7]. For example, restricted visitation inhibits communication with health care providers and can cause emotional distress for family members [8]. Thus, many studies have supported open patient visitation to meet family needs, prevent emotional distress, and improve the satisfaction of care [9,10].

However, an inverse policy has recently been implemented for family visitation. For example, Korea, which had the second largest number of confirmed Middle East respiratory syndrome (MERS) cases in the world after Saudi Arabia in 2015 [11], established strict regulations for infection control in medical institutions, including strong restrictions on family visitation at the hospital. The cultural customs of patient visitation and family caregiving are recognized as the main reasons why Korea initially failed to control the MERS outbreak [12]. Another example of such a policy shift relates to the COVID-19 pandemic. In particular, many countries have implemented administrative orders and quarantine guidelines to maintain social distancing and intercity travel restrictions for infection control. Medical institutions restricted people from visiting patients at hospitals to prevent the spread of COVID-19. According to a UK national survey [13], 117 (100%) hospitals reported that the family face-to-face visit policy in ICUs changed during the surge of COVID-19 cases; 19 (16%) hospitals reported no face-to-face family visits under any circumstances and 63% of hospitals indicated allowing family presence in certain circumstances such as at the end of life. Many hospitals still restrict family member visits to nonface-to-face visits [14]; consequently, several patients died without seeing their loved ones due to continuous social distancing and border closures [15,16].

Given a visit restriction policy implemented for patients in medical institutions worldwide, additional efforts are needed to prevent the negative consequences of these restrictions [6]. To reduce the negative impact, medical institutions have been using alternative communication technologies and platforms to conduct telephone calls and teleconferencing [5,6,8,17]. However, very few studies have explored whether such nonface-to-face visits have effects similar to those of face-to-face visits. Therefore, it is necessary to comprehensively review studies on nonface-to-face family visits through a systematic approach for generating evidence [18]. The purpose of this review was therefore to comprehensively explore nonface-to-face visits as an alternative during infection prevention and to synthesize the scientific evidence.

## Methods

### Search Strategy

A comprehensive search was conducted via the following five electronic databases up to September 10, 2021: PubMed, Embase, CINAHL, Cochrane, and Web of Science. In addition, we searched the clinical trials register ClinicalTrials.gov and Virginia Henderson International Nursing Library for unpublished trials up to the same date. The search query was developed according to the guidelines of the Peer Review of Electronic Search Strategies [19] and included keywords on the topics of telemedicine and visitation restrictions. The following search terms were used: telemedicine OR mobile health OR mhealth OR telehealth OR ehealth AND family AND visit. The search had no restrictions with respect to publication date or research design. Manual searches were performed via Google Scholar based on a reference list compiled from the articles retrieved from the above search strategy and databases for cross-referencing.

### Eligibility Criteria

**Figure 1:**
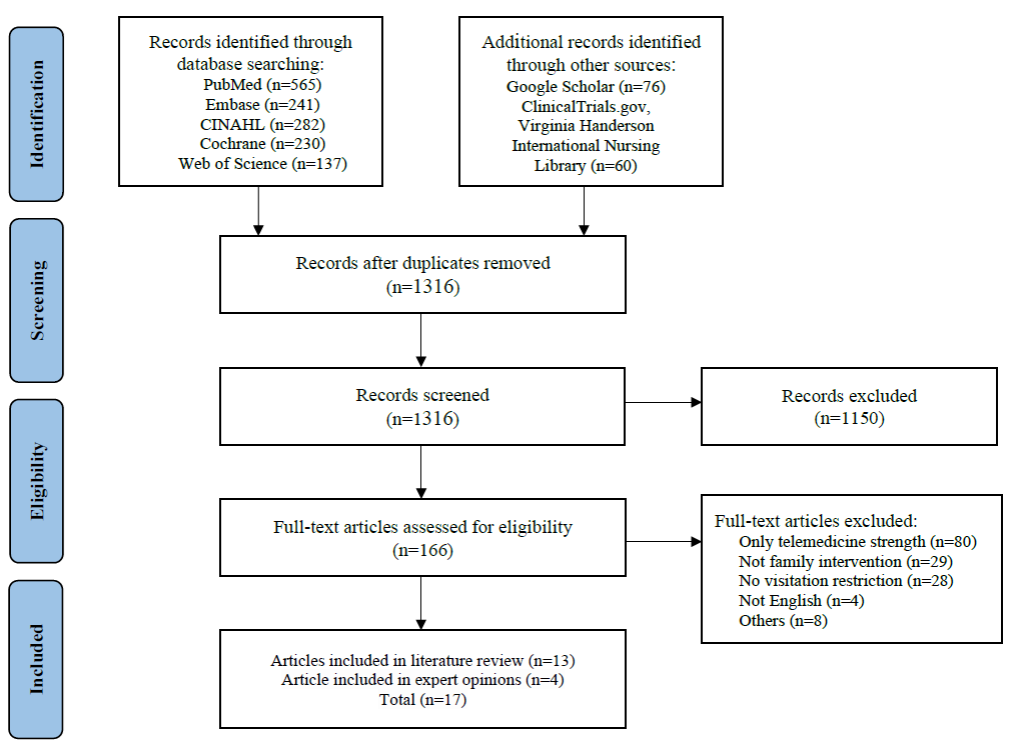
Flowchart of the data selection process. Others: No access to full-text article or other formats, such as editorials or reports.

The inclusion criteria were as follows: (1) nonface-to-face modality using telemedicine with family in a hospital setting (ICU or non-ICU ward), (2) experimental and observational studies, and (3) articles written in English. Exclusion criteria were as follows: (1) studies without access to the full text, (2) studies not including patient or family involvement, and (3) the results included information on the protocol or only a discussion of pros and cons without associated data. Finally, we identified 17 studies as eligible according to the inclusion/exclusion criteria (see Figure 1).

### Selection Process

All three authors independently reviewed the retrieved studies throughout the selection process. The screening process was conducted by two authors (JH and CY) who independently extracted and cross-checked the literature using search queries. Data were extracted in the Covidence program (Melbourne, Australia), which is a web-based software that specializes in systematic reviews. Covidence allows researchers to import and screen citations and full-text articles, resolve conflicts, extract data using customizable forms, and export results in standardized formats. Using Covidence in the process of reviewing the study, the criteria for inclusion and exclusion can be continuously developed and shared with the research team. Moreover, random arrangement methods can be designated, including listing the articles in author order or in the most recent order, which can help to avoid systemic bias in reviewing studies.

The search results were exported from Endnote into Covidence for screening. Duplicates were then automatically identified and removed using the same software. Two authors (JH and CY) independently screened titles, abstracts, and the full text by applying the potential eligibility criteria using Covidence. Thus, the full text of 166 articles was reviewed and the results were discussed until there was acceptable interreliability between the reviewers (κ=0.9). The two authors fully reviewed the selected articles after developing definite eligibility criteria and showed over 95% agreement regarding the final selection of the articles. The third author (KH) served as the external validator when the analyses were conflicting and helped to reach the final agreement of the selection. At this stage, any ambiguous aspects were discussed until a consensus was reached.

### Data Extraction and Analysis

The synthesis of evidence focused on the outcomes of nonface-to-face visits implemented during patient visit restrictions reported in each article. Because the review included various types of studies such as randomized controlled trials (RCTs), qualitative studies, and quasiexperimental studies, the following categories were used to conduct an integrative review: (1) characteristics of selected studies, (2) participants, (3) types of telemedicine, (4) benefits, and (5) limitations. Data extraction was independently performed by two authors (JH and CY) and any discrepancies were resolved through discussion.

Verification was conducted by comparing the results of the data analysis with the original articles. After data extraction and evaluation of study quality, summary tables were constructed regarding the study aims (see Multimedia Appendix 1). A detailed description of the data screening process is shown in Figure 1.

### Risk of Bias and Quality Assessment

Among the 17 studies included for review, four were based on expert opinions and commentary; hence, quality assessment was not applicable to these studies. The remaining 13 studies were critically assessed for methodological quality using the Joanna Briggs Institute critical appraisal checklists [20] depending on study design such as RCTs, qualitative, cross-sectional, quasiexperimental, and cohort studies. The quality summary of each study was determined by integrating the contents. The quality of the 13 studies was scored according to 1 point for “yes” and 0 points for “no,” “not applicable,” and “not reported” for each item on the checklist. The quality of each study was assessed independently by two authors (JH and CY) and discrepancies were discussed and resolved by the third author (KH). A summary of the quality assessments for the 13 included studies is presented in Multimedia Appendix 2.

## Results

### Characteristics of Selected Studies

Among the 17 studies selected, there were five qualitative studies, six survey studies, two experimental studies, and four expert opinions. The majority of the studies were conducted in the United States (n=9) and published in 2020 and 2021 (n=15). In 13 studies, excluding the four studies based on expert opinions, the duration of the study period varied from 2 weeks to 2 years of follow-up. The number of study participants ranged between 20 and 367. Most studies included family members of patients in the ICU (n=10). Some studies (n=2) included families of patients in palliative care. Most studies were limited to parents or family members (11/13, 85%), and the remaining studies included some nurses or health care providers (2/13, 154%). A summary of the study objectives, study designs, study participants, and their characteristics is provided in Multimedia Appendix 1.

### Content and Quality Assessment According to Study Type

Since various research methods were used in the included studies, the contents were analyzed according to the research design. The most common methods of data collection were interviews (n=5) and cross-sectional surveys (n=4). All five qualitative studies were conducted in the form of interviews, with research questions focused on describing clinical virtual pathways for visitation and communication [14] and exploring users’ evaluation of telemedicine for patients or family members [15,16,21,22]. The six survey studies consisted of five cross-sectional studies and one prospective cohort study including patients and families belonging to a wide range of ages. The nonface-to-face virtual visits involved families of patients in the ICU [13,21-25]; pediatric patients, including newborns in the NICU [14,26-28]; and older adults in hospitals and long-term care facilities [29,30]. With the advent of the global COVID-19 pandemic in 2020, virtual visitation and nonface-to-face visitation were generally introduced, and most of these studies adopted a qualitative research and survey design.

There were two experimental studies in total: one RCT evaluating the role of online video visitations [24] and one study on changes in patients and their families through intervention with virtual visit programs [31]. The quality assessment revealed that the quality of cohort, RCT, and qualitative research studies was generally acceptable. Although the quality of one of the cross-sectional studies was poor, the overall quality of these studies was high. However, a study using a quasiexperimental research method [31] had a mid-level quality standard because it did not clearly report whether participants were included in similar comparisons, whether group differences were adequately explained in follow-up measures and measured in the same way, and the statistical analysis method used for measuring the results.

### Types of Nonface-to-Face Visitation

Only 13 studies were reviewed in terms of the types of nonface-to-face visitation methods analyzed, excluding the four studies based on expert opinions. These 13 studies were related to new applications or programs and existing platforms such as Webcam, FaceTime (Apple), Zoom, Skype, Cisco Webex, and Microsoft Teams [13,15,27,31,32]. Since patients in the ICU who are generally sedated and intubated do not have independent access to these nonface-to-face telemedicine technologies, the medical staff is required to be at the patient’s side to provide mobile devices; thus, a platform was developed to meet the needs of these patients, their families, and medical staff [22]. New virtual care platforms were developed targeting specific groups, including Chez NICU Home [14] for newborns, Sickbay [22,23] for patients in the ICU, and Family-Link for children and their parents [28]. These new programs or applications are summarized in Table 1. Some of the telemedicine platforms only involve online video visitation [21,24,27,29,30]. Other platforms were also featured in the included studies, such as WhatsApp [16], TouchAway [13], and HowRU [25].

**Table 1:** Characteristics of new programs or applications for nonface-to-face visits.

Platform	Purpose	Function or features	Target user
Chez NICU home	A secure web-based platform that provides a NICU^a^ family with the training and resources needed to actively participate in baby care	Allows NICU patients and families, regardless of location, to connect with health care providers, families, and community health professionals and participate in customized interactive parental education and treatment	NICU patients and their families
Sickbay	Supports virtual care and remote monitoring workflows	Physiological data monitoring, including vitality sign monitors such as ventilators and virtual rounding, and mentoring of health care providers	Patients in the ICU^b^
TouchAway	Connects patients, clinicians, and families	Provides patient-centric care using virtual communication, treatment pathways, remote patient monitoring, care plan management, and many other tools	Clinicians, older adults, hospital patients, hospital administrators
HowRU	Open, flexible virtual visits tailored to patient-family–centric care	Ensures the privacy, dignity, and security of patients and families; facilitates an open and flexible communication line that adapts to the needs of each patient and family	Patients, families, and residents
Family-Link	Family child protection services that allow parents to adjust their children’s devices	Various content restrictions, device usage time management, GPS phone search, educational app download	Parents, children, and adolescents

^a^NICU: neonatal intensive care unit.

^b^ICU: intensive care unit.

### Benefits of Nonface-to-Face Visitation

The commonly reported benefits of nonface-to-face visits were promoting the psychological and physical recovery of patients [13,24,28-30] and reducing the psychological distress of family members by connecting them to their loved ones [13,16,22,23,25-27,31,32]. These nonface-to-face visits allow family members to meet their loved ones and make informed decisions about follow-up or provide end-of-life care before the patient’s death [31]. Another advantage reported was increased collective interaction when patients and their families met over video calls, which could include a group of people at the same time rather than one-on-one phone calls, allowing them to experience more social group dynamics [32]. In addition, nonface-to-face visits can enhance communication between family and health care teams through these virtual technology platforms, allowing family members to participate in the patient’s treatment [14,15,21-23]. In particular, video calls may be superior to phone calls to convey a general impression of the patient’s condition [15,21,30].

Although no study directly confirmed infection control as a primary outcome, almost all of the included studies stated some beneficial aspects of infection control [13-16,21-25,28-33]. Restricting face-to-face visits can prevent the spread of the virus, protect vulnerable patients from infectious diseases, and reduce the potential impact of infections on organizational environments [13,14,16,21,22,25,28,29,31-33]. As needed, nurses in the hospital rooms were required to perform nonface-to-face visits during patient treatment–related tasks to reduce the exposure of infection [15,22]. A study in Iran conducted before COVID-19 [24] reported that face-to-face visits were restricted due to concerns such as infection risk, delayed patient rest, invasion of patient privacy, and obstruction of nursing care.

Nonface-to-face visits also affected health care providers. Nonface-to-face visits can reduce the burden on health care providers and improve employee work ethic [13,32]. However, an employee survey revealed that the physical presence of physicians in the virtual ICU decreased [32]. In addition, a new virtual treatment platform and guidelines for the standardization of treatment have been established to provide continuous education even after nonface-to-face visits to enhance patient-centered treatment [13,14]. Using existing communication methods such as phone calls and email can also further promote positive emotions [21,29].

It is also necessary to develop a new platform because medical staff use personal protective equipment (PPE) and are exposed to unnecessary risk of infection [22]. The nonface-to-face visits can reduce unnecessary exposure and the overuse of PPE by the medical staff when caring for patients with infectious diseases [15,16,22,23,25,31]. In addition, the established virtual ICU allows nurses to request a quick visual review from a doctor, who is not required to wear PPE outside the patient room [32]. These nonface-to-face visits also provide additional benefits to obtain a convenient consultation from other health care professionals such as extracorporeal membrane oxygenation specialists, cardiologists, and other specialists, without physical contact [23].

### Limitations of Nonface-to-Face Visitation

Since face-to-face visitation restrictions come with barriers to effectively understanding and making decisions about the seriousness of the patient’s disease, it is always necessary for health care providers to use statements that can empathize with the family’s potential shock at seeing the potentially deteriorated state of the patient and warn the families accordingly before starting a nonface-to-face visit [15,32]. Most of the families who participated in nonface-to-face visitation shared positive emotions. However, some family members felt negative emotions, as they were sad to see the patient’s critical condition; however, these should not be interpreted as negative emotions about the nonface-to-face visit technology specifically [22,32]. Owing to the unpredictability of bedside nursing, it is impossible to reserve a nonface-to-face visit in advance. Hence, it is necessary to explain to the families in advance that the reservation may be canceled or stopped if urgent patient treatment is required since the family may not always be familiar with these aspects [22].

Media-specific difficulties of nonface-to-face telemedicine technology were also reported in the reviewed studies. Common barriers to nonface-to-face visits include technical problems such as network connection problems [13,22], access to appropriate devices [13,22,29,30,32], lack of staff time [13,22], potential for increased workload [13,14], and insufficient visiting time [31]. There are also problems associated with the effective use of nonverbal communication, including silence, limited facial expression, and difficulties in important discussions [21,22]. Specific concerns were raised about the lack of physical contact for patients in the pediatric ward who are separated from their parents [26-28]; however, no studies evaluated the effect of a lack of physical contact, and only one study reported a greater reduction in stress for children and parents who used nonface-to-face visits compared to those who did not [28].

Application of the latest technology is emphasized to protect medical staff and patients’ families from being in contact with infected patients as a part of patient-centered care in the pandemic era [23]. Although Facetime and Zoom can be used in everyday environments, patients in the ICU who are intubated and sedated cannot independently access these systems. Thus, medical staff must provide and operate mobile equipment to connect these patients to their families. If the patients do not have direct access to a platform for communication, there are privacy concerns about the sharing of personal information in the process of seeking external help [25]. Privacy protection may be difficult because of unintentional exposure of the medical staff to personal conversations between patients and their families in the ICU [32].

## Discussion

### Principal Results

This integrative literature review provides a timely understanding of virtual nonface-to-face visits for inpatient care. Our study summarizes the advantages and disadvantages of nonface-to-face virtual visits when face-to-face visits of families are impossible due to the risk of infection. The key findings from the included studies highlight the emotional distress experienced by patients and their families, such as isolation and loneliness, due to restrictions on face-to-face visits. Virtual nonface-to-face visits have been conducted using a variety of systems and platforms, with effects on patients, their families, and health care providers. Considering the characteristics of users and available technology, virtual nonface-to-face visits have become an important communication alternative with both advantages and disadvantages.

### Comparison With Prior Work

Since the outbreak of the COVID-19 pandemic, a nonface-to-face approach has been adopted worldwide in many areas such as education, health care, and business. Previous studies have reported that nonface-to-face family visits protect health care providers and reduce PPE use while providing treatment [34]. Similar to the 2009 H1N1 influenza pandemic and the 2014 MERS epidemic, hospital transmission should be reduced through the use of protective equipment for visitors and staff, hand hygiene, and proper precautions [35,36]. Restricting patient visits has become an axiom-based public health policy to maximize the benefits for the community [5]. Strict isolation and visitation restrictions protect vulnerable patients from infection and reduce the risk of infection to their families and health care providers who care for infected patients [37,38]. Specifically, infection of health care providers causes a workforce shortage, increasing the burden on remaining health care providers in a pandemic [39].

Nonface-to-face visits have been proposed as an alternative to support patients and their families, most significantly since the outbreak of the COVID-19 pandemic with advances in associated technology. Virtual visitation aids in meeting the patient and communicating with the medical team without physical contact. To reduce the negative consequences of patient isolation, access to various social technologies has been widely proposed and the use of telemedicine services has increased [21,30]. However, it has been difficult to cope with the unexpected demise of loved ones [15] and some report a struggle with unfamiliar communication methods [16,29]. For example, the studies on video technology–based interventions such as FaceTime and Skype have shown parental appreciation for being able to see their baby when the NICU is inaccessible [40]. However, a few parents felt guilty for not being able to stay with their children when they met them virtually [41]. Similarly, families of severely ill patients felt negative emotions such as sadness when they observed the serious situation on video [15,32]. In addition, the type of support or demand received after the video session is unknown [15], and families indicated that it is confusing to ask medical providers about the health status or treatment process of the patient due to concerns related to the unpredictable nature of a disease course [22]. Families should be able to visit patients whenever they wish; however, the importance of communication is emphasized over visits because of the priority accorded to patients [42].

There are several potential ways to expand communication with the medical team that continuously informs family members about the patient’s current state or to help with emotional conversations and provide support to the patients. Health care providers are also grateful for being able to emotionally help patients in difficult-to-face situations and acknowledged that these were crucial interactions [43]. In a situation where face-to-face visits were completely banned, ICU medical teams were encouraged to form a bond with the patients’ families and act as mediators between the patients and their families [44]. However, there was a difference in perspective between the family and the medical team: while families wanted to communicate with medical staff more regularly and frequently, clinicians were responsible for managing nonface-to-face virtual visits, which was perceived as a heavy emotional burden [21,45].

Furthermore, prohibiting or restricting visitors has raised the ethical issues of exercising the right to freedom and not being able to see family members. Some studies expressed concern about the impact that such restrictions have on the bond between pediatric patients and their families, suggesting that the potential risk of infection should be weighed against the adverse effects of visit restrictions [46]. There is a conflict between the demands of the patient’s family when they want to see the patient and the medical staff who wish to manage the patient’s treatment. Finally, the health care provider who is close to a patient can inflict moral damage by invading their privacy or eavesdropping on the intimate and emotional conversations between the patient and family in the process of helping with nonface-to-face visits [32].

### Implications for Research

This review provides a comprehensive understanding of the effects of nonface-to-face virtual visits for patients, their families, and medical staff; however, only one study was conducted based on a rigorous research design such as an RCT. Thus, more studies are required to examine the effectiveness of nonface-to-face virtual visits on patient outcomes and care team performance in diverse care settings with larger samples. In this review, the data were synthesized and presented descriptively along with context from other studies, including articles based on expert opinions. This is a methodological limitation, and further research will require more rigorous methodologies such as using probability sampling, controlling for confounding variables, and focusing on a narrow range of subjects such as patients in critical conditions and the NICU to increase the likelihood of the generalization of results.

Most studies included in this literature review were conducted in developed countries such as the United States, the United Kingdom, and Australia. This delineates the limitation of differences in accessibility to virtual visits and telemedicine in different countries. Since nonface-to-face and virtual visits are not limited to geographic areas, it is considered necessary to apply various research methodologies and to conduct more follow-up research on various patient groups and families by country and culture. A wide range of large-scale studies is needed, including various countries and institutions and patients of varied ages and with different diseases. Different countries have varied accessibility to virtual nonface-to-face systems, which may lead to a cultural difference in their understanding of visits; thus, our results may not be appropriate for generalization.

Experienced multidisciplinary medical staff can strengthen positive attitudes toward patients and their families to overcome the shortcomings of nonface-to-face virtual visits. This offers the potential for integrated development of a research-education-theory pathway, as the initial idea of research has begun in practice. It is further necessary to develop appropriate programs with the help of user-friendly technologies for nonface-to-face virtual visits. To supplement these virtual visits, ensuring the availability of technology is necessary. Future recommendations are proposed to expand the number of electronic devices and employees, as well as to simplify communication technologies to improve programs and platforms and extend allocated nonface-to-face visits [30,31].

A theory-based study is needed to explain health-related behaviors using nonface-to-face virtual smartphone apps or digital electronic devices. In this review, the complexity of communication technology and difficulty in accessing devices were reported as disadvantages of nonface-to-face visits; thus, efforts to simplify them are needed. As it is more difficult for older people to use new technologies and devices than young people, a systematic theoretical framework is needed to consider digital literacy and develop reliable and effective tools so as not to distort the actual meaning of the results.

We suggest measures to ensure the sustainability of these digital solutions. There is a need to expand facilities that allow digital solutions such as teleconferencing, telemedicine, and nonface-to-face visits to be implemented without direct contact. In addition, training on digital technology and equipment is required, and systematic education is also needed for patients, caregivers, and families. To continue using these digital solutions, privacy regulations and policies for personal information protection should be established so that the technology can be used without invading personal privacy. In fact, the biggest critique is that these digital solutions can become “fun and expensive toys.” Therefore, continuous verification is required to ensure that the platforms are truly therapeutic and to optimize by whom, when, how, and how long they should be used to achieve an appropriate treatment effect; only then can a clinical protocol be presented. This can be followed by an economic evaluation such as the cost of the interface or decrease in the prescription of anxiolytics.

### Implications for Education and Practice

The family’s role as a gatekeeper is important for nonface-to-face virtual visits [25]. It is important to find a family-centered approach, guide the family to maximize virtual visits, and improve their understanding of the family experience through telephone and video communication [13,21]. The family can have direct access to the patient, improve the technical function of the system to use technology that simulates the family’s face-to-face visit experience, improve the visiting process, and allow for more frequent communication between families and health care providers [21,22]. In particular, proficient use of platforms or mobile apps has a great influence on the results of interventions using virtual and telehealth technologies. To overcome the shortcomings of nonface-to-face virtual visits and enhance their strengths, educational opportunities to learn about multimedia devices, technologies, and mobile phones should be provided in hospitals. Education on these technologies is indispensable and can be further expanded to experience in other fields in the future.

Health care providers expressed that remote nonface-to-face visits were not a completely new communication strategy but rather a modification of existing communication strategies [21]. A study related to communication types and emotional experiences emphasized the synchronicity of communication, reporting that more frequent calls were associated with less negative emotional experiences and more positive perceived experiences [30]. In addition, a high level of satisfaction was associated with both video and telephone calls and the satisfaction level of video calls increased even more with the help of technology. Thus, it is necessary to select a communication type that suits the characteristics of the participants. Only 30% of medical institutions provide employee education for family communication and virtual visits [13]. Since virtual visits are widespread during the pandemic, it is necessary to expand employee training.

Some of the extracted literature included discussion of efforts toward protecting the private data of the patients and families during virtual visits [13,21,23,27,32]. Specific methods have been implemented by controlling the spread of webcam passwords [27], using “clean” iPads [32], or using proprietary software limited by concerns about the security of the system [13,32]. Other activities to secure privacy include (1) one-way calls only initiated by the medical team, (2) using secure cloud-based storage, (3) setting up two-step authentication for virtual visits, and (4) avoiding the use of personal devices by the medical team [13]. In addition, researchers also emphasized the expansion of video communication software [32], administrative and institutional support [21], and customization to comply with the Health Insurance Portability and Accountability Act [21,23]. Thus, the approach to security should take into account ease of use so that it works for users [47]. Therefore, medical team practitioners should value data security and software developers need to develop new technologies for user-friendly security systems.

### Strengths and Limitations

The advantages of this review were that each process of the research—keyword identification, extracted studies, quality assessment, and analysis—ensured independence and consistency while using the Covidence program, which helped to standardized the review. Moreover, this study is closely related to practices utilizing research team clinical experience in the ICU and other special units in the hospital.

There are also several limitations to this study. First, there are few experimental studies to ensure the effectiveness of virtual visitation on diverse patient outcomes. Second, many studies on nonface-to-face and virtual interventions, telemedicine, and smartphone app development have been reported in a short period due to visit restrictions and social distancing recommendations in the COVID-19 era. As research results related to nonface-to-face medicine, telemedicine, and digital therapeutics are reported explosively during this period, many studies that meet the criteria for this review but were reported after the search period may have been excluded. Third, there were many potentially eligible studies in the grey literature, some of which were reported only as conference abstracts in posters. In addition, it is impossible to measure effect size or perform statistical pooling in qualitative studies at this point and there is a lack of information to conduct a systemic review and meta-analysis.

### Conclusions

Despite its limitations, this study provides important information about patients, their families, and medical staff for nonface-to-face virtual visits. Face-to-face visit limitations caused emotional distress for the families of hospitalized patients and nonface-to-face virtual visits that made up for these restrictions helped to lower the risk of infection for the patient and family. Virtual visits also facilitated the interaction between patients and their families and helped families participate in the patient’s care by communicating with medical staff. This promoted recovery on both psychological and physical levels, while decreasing psychological distress. However, technical issues with networks and devices are reported as limitations and nonface-to-face virtual technology could not replace the actual presence of families. Ensuring the availability of technology and educating on the same in alignment with the characteristics of patients and their families, nonface-to-face virtual visits need to show more potential as an effective patient-centered treatment strategy based on more research and advanced practice.

## Appendix

Multimedia Appendix 1 Summary of characteristics of articles and methodological approaches from 17 studies.

Multimedia Appendix 2 Methodological evaluation of study quality.

Multimedia Appendix 3 PRISMA checklist.

## Abbreviations

ICU: intensive care unit
MERS: Middle East respiratory syndrome
NICU: neonatal intensive care unit
PPE: personal protective equipment
RCT: randomized controlled trial

## Data Availability

All data analyzed during this study are included in Multimedia Appendix 1.
