# Supplementary material for: Nonface-to-Face Visitation to Restrict Patient Visits for Infection Control: Integrative Review
Source: Interact J Med Res. 2023 Nov 28;12:e43572. doi: 10.2196/43572 (PMC10686251; doi:10.2196/43572)
Supplement: Multimedia Appendix 1 [file ijmr_v12i1e43572_app1.docx]

## Multimedia Appendix 1. Summary of characteristics of articles and methodological approaches from 17 studies

| Study, year  (country) | Design and sample | Participants | Telemedicine | Duration | Key points |
| --- | --- | --- | --- | --- | --- |
| Rose et al, 2021(UK) [13] | A cross-sectional study, 117 | UK hospitals with  at least one ICU. | Virtual visiting (ex. aTouchAway, Skype, FaceTime, Zoom, etc..) | 1 month | Virtual visiting and dedicated communication teams were common COVID-19 pandemic innovations addressing the restrictions to family ICU visiting, and they resulted in valuable benefits in terms of patient recovery and staff morale. Enhancing access and developing a more consistent approach to family virtual ICU visits could improve the quality of care, both during and outside of pandemic conditions. |
| Campbell-Yeo et al, 2021(Canada) [14] | Qualitative interviews, 20 | parent partners, neonatal HCPs, administrators, and researchers | A new virtual care platform. Chez NICU Home© (CNH) | 6 months | The development of the clinical care pathways was done through an agile, collaborative process based on the needs identified from NICU families and HCPs. To enact meaningful change, decisions on the format of these pathways were responsive to the needs of families and pragmatic in considering current unit practices. |
| Kuntz et al, 2021(USA) [15] | Interviewing study, 63 | family participants | Zoom | 26 days | We demonstrated the efficient deployment of telemedicine for e-family meetings that was both feasible and effective for decision-making for patients who were near the end of life and their families. Family meetings likely happened sooner and with far more participants than would have been possible without the use of the technology. |
| Mercadante et al, 2021(Italy) [16] | Interviewing study, 16 | family members | WhatsApp | 2 weeks | WhatsApp can help patients and their families. Most family members had a good impression. However, the real presence bedside was considered irreplaceable. |
| Kennedy et al, 2021(USA) [21] | A qualitative interviewing study, 35 | 21 family members and 14 treating clinicians | phone and video  interactions | 1 month | Telehealth communication between families and clinicians of ICU patients appears to be a somewhat  effective alternative when in-person communication is not possible. The use of communication strategies specific to phone and video can improve clinician and family experiences with telehealth. |
| Sasangohar et al, 2021(USA) [22] | Qualitative analysis, 230 | family members of ICU patients | Sickbay and Consultant Bridge | 3 weeks | We documented our novel and improvised adoption of the virtual ICU technology to enable family visitation and explore the feedback we received. While the overall experience of family members was overwhelmingly positive, several key barriers, concerns, and areas for improvement were identified. Given the promise shown by this successful implementation, recent emphasis on involving family members in care. |
| Dhala et al, 2020(USA) [23] | Expert’s opinions, no mention | no mention | Sickbay and Consultant Bridge | no mention | Telecritical care has been established as an advantageous mechanism for the delivery of critical care expertise. The current COVID-19 pandemic has brought multiple new useful applications to light that could be transformative in how telecritical care is perceived and deployed in the future, especially during highly infectious disease outbreaks. |
| Shahdosti et al, 2020(Iran) [24] | RCT, 66 | CABG candidates | online video | 7 months | Online video visitations decreased anxiety in patients at ICU-OH. Since these patients experience strict restrictions on visiting family members, online video visitations can be a suitable replacement for in-person visits. In addition to the mental support of patients by their families, online video visitations are easy to use and cost-effective and decrease referrals to hospitals. |
| Thomas et al, 2021(Australia) [25] | Expert’s opinions, no mention | family member | HowRU and Cisco Webex | No mention | we can now provide open and flexible virtual visiting that aligns with patient-family-centered care using HowRU. HowRU aligns with our usual code of conduct, ensuring patient and family privacy, dignity, and security. It facilitates an open and flexible line of communication that can be adapted to the needs of each individual patient and family. |
| Chheda and Leiner, 2021(USA) [26] | A cross-sectional study, no mention | NICU parents and nurses. | Virtual visitation (no mention of telemedicine) | no mention | Even though families have described baby interactions as important to reducing stress levels and feelings of anxiety, and to developing parent–infant attachment, the virtual visitation can become another tool to help parents reduce the stress caused by the physical separation. |
| Thibeau et al, 2012(USA) [27] | Expert’s opinions, no mention | NICU family | Webcam | No mention | Innovative technology such as webcams offers virtual visitation, which holds the potential to improve parent-infant bonding and reduce parents’ self-perceived stress. |
| Yang et al, 2014(USA) [28] | A prospective cohort study, 367 | pediatric patients | Family-Link | 28 months | The use of videoconferencing by some hospitalized children and families to conduct virtual visits with family and friends outside of the hospital was associated with a greater reduction in stress during hospitalization than those who did not use videoconferencing. |
| Monin et al, 2020(USA) [29] | A cross-sectional study, 161 | Community-dwelling adults who had a family member or friend in a Long-Term Care facility. | nine communication methods (phone, video-conference, e-mail, and letters) | 34 days | Connecting with family members and friends in long-term care facilities, especially via phone, may contribute to better emotional experiences for family members, friends, and long-term care residents. |
| Sacco et al, 2020(France) [30] | A cross-sectional study, 132 | Older hospital patients and nursing home residents | Telephone calls and video calls | 6 weeks | We found that older adults confined to healthcare settings were more often independently able to perform telephone calls than video calls, and they tended to use the telephone more often to communicate with their relatives. |
| Mendiola et al, 2021(USA) [31] | Experimental study, 54 | patients | Microsoft Teams | 24 days | A virtual visitation program was implemented in critical care units to replicate visitation by video chat to ease the stress on patients and family members to improve communication. |
| Webb et al, 2020(UK) [32] | Survey study, 45 | Medical staff (physiotherapists, nurses, healthcare assistants, and doctors) | Facetime | 3 months | We report on measures introduced in response to this situation at the critical care unit in one hospital involving the use of videoconferencing technology. The solutions used also had the potential to be extended to clinical use, for example when seeking advice for colleagues, and to the provision of training. |
| Voo et al, 2020(Singapore) [33] | Expert’s opinions, no mention | No mention | e-communication infrastructure (no mention of telemedicine) | No mention | Virtual presence for isolated patients is often recommended and used to enable communication. We consider the ethical management of these modes of family presence and argue for the promotion of physical presence under some conditions. |
