# Supplementary material for: Nonface-to-Face Visitation to Restrict Patient Visits for Infection Control: Integrative Review
Source: Interact J Med Res. 2023 Nov 28;12:e43572. doi: 10.2196/43572 (PMC10686251; doi:10.2196/43572)
Supplement: Multimedia Appendix 2 [file ijmr_v12i1e43572_app2.docx]

## Multimedia Appendix 2. Methodological evaluation of study quality

JBI CRITICAL APPRAISAL CHECKLIST FOR ANALYTICAL CROSS-SECTIONAL STUDIES

|  |  | Rose et al, 2021 (UK) [13] | Chheda and Leiner, 2021 (USA) [26] | Monin et al, 2020 (USA) [29] | Sacco et al, 2020 (France) [30] | Webb et al, 2020 (UK) [32] |
| --- | --- | --- | --- | --- | --- | --- |
| 1 | Were the criteria for inclusion in the sample clearly defined? | 1 | 0 | 1 | 1 | 0 |
| 2 | Were the study subjects and the setting described in detail? | 1 | 1 | 1 | 1 | 1 |
| 3 | Was the exposure measured in a valid and reliable way? | 1 | 0 | 1 | 1 | 0 |
| 4 | Were objective, standard criteria used for measurement of the condition? | 1 | 0 | 1 | 1 | 0 |
| 5 | Were confounding factors identified? | 1 | 0 | 1 | 1 | 1 |
| 6 | Were strategies to deal with confounding factors stated? | 1 | 0 | 1 | 1 | 1 |
| 7 | Were the outcomes measured in a valid and reliable way? | 1 | 0 | 1 | 1 | 0 |
| 8 | Was appropriate statistical analysis used? | 1 | 0 | 1 | 1 | 0 |
| Sum |  | Include | Include | Include | Include | Include |

JBI CRITICAL APPRAISAL CHECKLIST FOR QUALITATIVE RESEARCH

|  |  | | Campbell-Yeo et al, 2021 (Canada) [14] | | Kuntz et al, 2021 (USA) [15] | | | Mercadante et al, 2021 (Italy) [16] | | | Kennedy et al, 2021 (USA) [21] | Sasangohar et al, 2021 (USA) [22] | | |  |  |
| --- | --- | --- | --- | --- | --- | --- | --- | --- | --- | --- | --- | --- | --- | --- | --- | --- |
| 1 | Is there congruity between the stated philosophical perspective and the research methodology? | | 0 | | 1 | | 1 | | | 1 | | | | 1 | | |
| 2 | Is there congruity between the research methodology and the research question or objectives? | 1 | | 1 | | 1 | | | 1 | | | | 1 | | |  |
| 3 | Is there congruity between the research methodology and the methods used to collect data? | 1 | | 1 | | 1 | | | 1 | | | | 1 | | |  |
| 4 | Is there congruity between the research methodology and the representation and analysis of data? | 1 | | 1 | | 1 | | | 1 | | | | 1 | | |  |
| 5 | Is there congruity between the research methodology and the interpretation of results? | 1 | | 1 | | 1 | | | 1 | | | | 1 | | |  |
| 6 | Is there a statement locating the researcher culturally or theoretically? | 0 | | 0 | | 1 | | | 0 | | | | 0 | | |  |
| 7 | Is the influence of the researcher on the research, and vice-versa, addressed? | 0 | | 1 | | 0 | | | 1 | | | | 1 | | |  |
| 8 | Are participants, and their voices, adequately represented? | 0 | | 1 | | 1 | | | 1 | | | | 1 | | |  |
| 9 | Is the research ethical according to current criteria or, for recent studies, and is there evidence of ethical approval by an appropriate body? | 1 | | 1 | | 0 | | | 1 | | | | 1 | | |  |
| 10 | Do the conclusions drawn in the research report flow from the analysis, or interpretation, of the data? | 1 | | 1 | | 1 | | | 1 | | | | 1 | | |  |
| SUM |  | Include | | Include | | Include | | | Include | | | | Include | | |  |

JBI CRITICAL APPRAISAL CHECKLIST FOR QUASI-EXPERIMENTAL STUDIES

|  |  | Mendiola et al, 2021 (USA) [31] |
| --- | --- | --- |
| 1 | Is it clear in the study what is the ‘cause’ and what is the ‘effect’ (i.e. there is no confusion about which variable comes first)? | 1 |
| 2 | Were the participants included in any comparisons similar? | 0 |
| 3 | Were the participants included in any comparisons receiving similar treatment/care, other than the exposure or intervention of interest? | 1 |
| 4 | Was there a control group? | 1 |
| 5 | Were there multiple measurements of the outcome both pre and post the intervention/exposure? | 1 |
| 6 | Was follow up complete and if not, were differences between groups in terms of their follow up adequately described and analyzed? | 0 |
| 7 | Were the outcomes of participants included in any comparisons measured in the same way? | 1 |
| 8 | Were outcomes measured in a reliable way? | 0 |
| 9 | Was appropriate statistical analysis used? | 0 |
| SUM |  | Include |

JBI CRITICAL APPRAISAL CHECKLIST FOR RANDOMIZED CONTROLLED TRIALS

|  |  | Shahdosti et al, 2020 (Iran) [24] |
| --- | --- | --- |
| 1 | Was true randomization used for assignment of participants to treatment groups? | 1 |
| 2 | Was allocation to treatment groups concealed? | 1 |
| 3 | Were treatment groups similar at the baseline? | 1 |
| 4 | Were participants blind to treatment assignment? | 1 |
| 5 | Were those delivering treatment blind to treatment assignment? | 0 |
| 6 | Were outcomes assessors blind to treatment assignment? | 0 |
| 7 | Were treatment groups treated identically other than the intervention of interest? | 1 |
| 8 | Was follow up complete and if not, were differences between groups in terms of their follow up adequately described and analyzed? | 1 |
| 9 | Were participants analyzed in the groups to which they were randomized? | 1 |
| 10 | Were outcomes measured in the same way for treatment groups? | 1 |
| 11 | Were outcomes measured in a reliable way? | 1 |
| 12 | Was appropriate statistical analysis used? | 1 |
| 13 | Was the trial design appropriate, and any deviations from the standard RCT design (individual randomization, parallel groups) accounted for in the conduct and analysis of the trial? | 1 |
| SUM |  | Include |

JBI Critical Appraisal Checklist for cohort studies

|  |  | Yang et al, 2014 (USA) [28] |
| --- | --- | --- |
| 1 | Were the two groups similar and recruited from the same population? | 1 |
| 2 | Were the exposures measured similarly to assign people to both exposed and unexposed groups? | 1 |
| 3 | Was the exposure measured in a valid and reliable way? | 1 |
| 4 | Were confounding factors identified? | 1 |
| 5 | Were strategies to deal with confounding factors stated? | 1 |
| 6 | Were the groups/participants free of the outcome at the start of the study (or at the moment of exposure)? | 1 |
| 7 | Were the outcomes measured in a valid and reliable way? | 1 |
| 8 | Was the follow up time reported and sufficient to be long enough for outcomes to occur? | 1 |
| 9 | Was follow up complete, and if not, were the reasons to loss to follow up described and explored? | 0 |
| 10 | Were strategies to address incomplete follow up utilized? | 0 |
| 11 | Was appropriate statistical analysis used? | 1 |
| sum |  | Include |
